# Periodic Noise Characteristics and Acoustic Control in Long Highway Tunnels: An FEM Study with In Situ Validation

**DOI:** 10.3390/ma19081548

**Published:** 2026-04-13

**Authors:** Ruifeng Ding, Xingyu Gu, Chenlin Liao, Hongchang Wang, Zengbin Xu, Kaiwen Lei, Jiwang Jiang

**Affiliations:** 1 Xiamen Municipal Transportation Construction Quality and Safety Center, Xiamen 361001, China; 18905923706@163.com; 2 School of Transportation, Southeast University, Nanjing 211189, China; 3 Xiamen R&B BAICHENG Co., Ltd., Xiamen 361009, China

**Keywords:** tunnel noise, acoustic finite element model, sound pressure level, noise mitigation, sound-absorbing panels, porous asphalt pavement

## Abstract

Noise in long highway tunnels and underground interchanges poses a significant environmental concern, affecting both drivers and nearby residents. This research develops an acoustic finite element model of a long tunnel in Leuven Measurement Systems (LMS) Virtual Lab to characterize the tunnel noise field, and the effectiveness of different noise mitigation measures was also evaluated and optimized accordingly. The model is validated against in situ monitoring data, with deviations controlled within 3 dB(A) and strong agreement confirmed by the Kappa consistency test. Both simulations and measurements show that sound pressure levels (SPLs) are generally highest near the tunnel center and lower toward the portal, exhibiting periodic fluctuations rather than a monotonic decrease. The dominant noise energy is concentrated between 125 Hz and 500 Hz. SPLs at 1.8 m above the road surface are noticeably higher than at 1.2 m and 1.5 m, indicating greater noise exposure for drivers of large vehicles compared with smaller vehicles. Noise reduction performance is further assessed for different lining materials and pavement types. Installing sound-absorbing panels in the tunnel midsection provides effective attenuation, with expanded perlite panels, single-layer metal micro-perforated panels, and FC quiet perforated panels (FC-PP) performing best, while porous asphalt shows superior noise reduction compared with conventional dense-graded asphalt pavements.

## 1. Introduction

With the rapid development of China’s highway network, a large number of tunnels and underground interchanges have been built. Traffic noise in tunnels is affected by vehicle type, tunnel geometry, and surface materials [1,2,3]. Due to the enclosed space, noise tends to accumulate and reflect, making noise levels inside tunnels and at portals much higher than those on open roads [2,4]. This not only reduces ride comfort and increases driver fatigue but also affects the living environment of nearby communities [1,5,6]. In some cases, tunnel noise levels even exceed permissible limits, posing potential health and safety risks [1,5]. These issues highlight the need to better understand tunnel noise characteristics and develop effective noise reduction strategies.

Research on tunnel noise has mainly relied on field monitoring, numerical simulation, and analytical evaluation. Field monitoring provides direct information on noise levels and frequency distributions [1,2,7]. Numerical simulations, particularly finite element modeling [8], can be used to reproduce tunnel acoustic fields and evaluate their responses under different traffic scenarios [8,9,10]. Three-dimensional and hybrid numerical methods have also been applied in related enclosed-transport acoustic studies [10,11]. Overall, these studies indicate that tunnel-noise analysis has developed into a relatively mature methodological framework. Early full-scale investigations demonstrated that coherent sound-field models can reproduce tunnel noise transmission with good accuracy and outperform simplified energy-based approaches, especially in the low-frequency range where interference effects are significant [4]. At a broader scale, strategic noise mapping and harmonized assessment frameworks such as the Environmental Noise Directive and CNOSSOS-EU have improved the consistency and comparability of road-traffic noise evaluation [6,12]. In parallel, data-driven methods, including neural-network-based models and probabilistic models derived from short-term monitoring data, have further expanded the available approaches for traffic-noise prediction [13,14]. More recently, tunnel-oriented studies have combined field measurements with numerical simulation to optimize low-noise pavements and sound-absorbing devices, confirming that both pavement design and absorptive treatments can significantly improve the acoustic performance of tunnel environments [2,7,15]. These studies provide an important foundation for tunnel-noise analysis and mitigation planning.

Building on these developments, researchers have explored both active and passive noise control strategies. Although active noise control has shown potential for reducing low-frequency noise [16,17], its complexity and high cost limit its application in highway tunnels [17]. In practice, passive measures such as noise-reducing pavements and sound-absorbing panels are more commonly adopted [3,7,15]. However, existing studies have mainly focused on design-stage prediction, controlled acoustic experiments, or the evaluation of individual mitigation measures, whereas post-evaluation studies on in-service road tunnels remain relatively limited [1,2,7,8,9,10,15,16,17]. One major reason is the lack of sufficiently detailed field data under real operating conditions, including traffic characteristics, spatial noise distributions, and local boundary information, which are essential for reliable model calibration and validation. In other words, although traffic-noise simulation and acoustic prediction methods have become increasingly mature, models that can accurately represent the actual noise state of existing tunnels under real traffic loading are still insufficient. This limitation also restricts the objective comparison and optimization of alternative noise-reduction measures for operating underground road facilities.

To address this gap, the present study proposes a data–model co-driven framework for the post-evaluation and optimization of noise mitigation in an existing road tunnel or underground interchange. First, multi-level field measurements are conducted to obtain real traffic-noise data and to analyze the influence of key factors, including traffic composition, vehicle speed, lane distribution, geometric position, and pavement characteristics, on measured noise levels. Second, an acoustic finite element model of the in-service tunnel under vehicle loading is established and calibrated using the field data to further reveal the spatial and spectral characteristics of the tunnel noise field. Finally, the measured data and the validated numerical model are jointly used to compare and optimize alternative noise-reduction measures, thereby providing a more realistic basis for the post-evaluation and optimized design of noise-control strategies in operating underground road systems.

## 2. Acoustic Finite Element Modeling and Measurement Methods

### 2.1. Development of the Tunnel Acoustic Model

#### 2.1.1. Finite Element Method for Acoustic Field Calculation

The tunnel acoustic field is described using the classical acoustic wave equation, which is derived from the linearized fluid continuity, momentum, and state equation. For a lossless, stationary fluid, the acoustic wave equation in the time domain is given by Equation (1) [18]:

(1) $$\nabla^{2}p-\frac{1}{c^{2}}\frac{\partial^{2}p}{\partial t^{2}}=-\rho_{0}\frac{\partial q}{\partial t}$$

where ρ is the acoustic pressure; $\rho_{0}$ is the density of the stationary fluid; c is the speed of sound; and q represents the acoustic source term. For frequency-domain analysis, applying the Fourier transform to Equation (1) yields Equation (2):

(2) $$\nabla^{2}p(x,y,z)-k^{2}p(x,y,z)=-j\rho_{0}\omega q_{0}(x,y,z)$$

where k=ω/c is the wavenumber; ω=2πf is the angular frequency; and f is the frequency.

To solve the acoustic field numerically, appropriate boundary conditions are applied. The particle velocity condition specifies the normal velocity on boundary nodes to capture structural vibrations, the pressure condition sets acoustic pressure on boundaries, and the impedance (mixed) condition links pressure and velocity to model material absorption or reflection.

The acoustic wave equation and boundary conditions are discretized on the finite element mesh to form a matrix Equation (3) [8]:

(3) $$(K_{a}+j\omega C_{a}-\omega^{2}M_{a})p_{i}=F_{i}$$

where $p_{i}$ is the nodal pressure vector; $K_{a}$, $C_{a}$ and $M_{a}$ are the acoustic stiffness, damping, and mass matrices, and $F_{i}$ includes source and boundary excitations. Solving this sparse matrix equation yields the acoustic pressure distribution inside the tunnel.

#### 2.1.2. Acoustic FEM Model of the Tunnel

**Figure 1:**
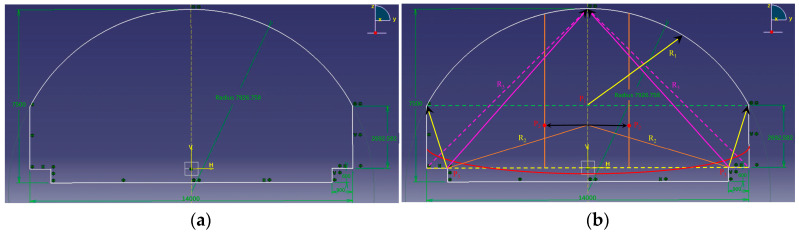
The cross-section of FEM model in Virtual Lab. (**a**) Simplified cross-section; (**b**) Detailed arrangement diagram of cross-section. Solid and dashed lines indicate different sound propagation paths, and arrows denote the direction of propagation. R1–R3 and P1–P3 represent characteristic paths and points, respectively.

In acoustic research, software such as SYSNOIS, Virtual Lab, and EASE has been widely adopted internationally. Among these, Virtual Lab provides a broader computational frequency range and strong compatibility with CAD platforms, making it particularly suitable for detailed tunnel acoustic analysis [8]. Previous studies have shown that, under appropriate boundary conditions and grid resolutions, acoustic simulations based on the finite element method can accurately reproduce the sound pressure distribution in tunnel environments [11]. In this study, the tunnel acoustic model was developed in Virtual Lab 13.6 based on the tunnel cross-section of an actual long tunnel in Xiamen City, as shown in Figure 1.

Following the construction of the tunnel model, the mesh is generated and defined as an acoustic mesh using the acoustic finite element module. The size and refinement of the mesh critically affect both computational efficiency and solution accuracy. In general, at least six elements are required within the shortest wavelength. Accordingly, the maximum element edge length should be less than one-sixth of the shortest target wavelength [8]. Given that the sound propagation speed in the medium is *c*, the element length is *L*, and the highest frequency of interest is *f*_max_, the element size must satisfy Equation (4):

(4) $$L\leq \frac{c}{6f_{max}}$$

Traffic noise is characterized as a broadband line source, with most of its acoustic energy concentrated in the low- and mid-frequency ranges, typically peaking around 63 Hz and 1000 Hz [7]. Preliminary simulations indicate that sound pressure levels above 2000 Hz are negligible compared to those around 1000 Hz, and high-frequency computations tend to amplify modeling errors.

**Figure 2:**
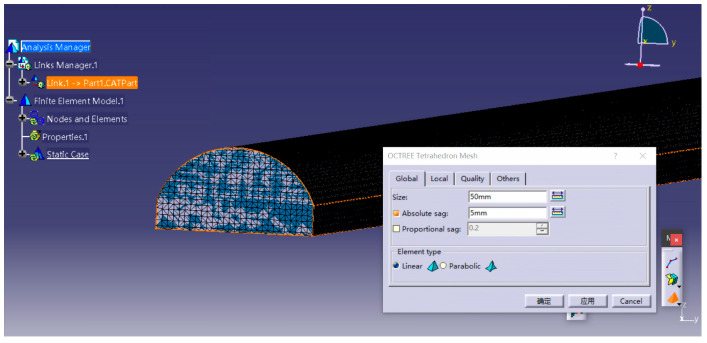
Meshing of the finite element model.

Therefore, the frequency range in this study was limited to 63–1000 Hz ($f_{max}$ = 1000 Hz). Substituting $f_{max}$ and *c* = 340 m/s into Equation (4) yields a maximum element size of 0.056 m. To balance computational efficiency and accuracy, the tunnel model was discretized using tetrahedral elements with an edge length of 50 mm. The mesh of the finite element model is shown in Figure 2.

#### 2.1.3. Material Parameters

In the tunnel environment, sound waves propagate through the air medium. When these waves encounter the tunnel walls or the pavement surface, a portion of the energy is reflected back into the tunnel, while the remaining portion is absorbed by the wall and pavement materials. Subsequently, the residual sound waves gradually propagate from the tunnel portals into the external atmosphere.

In the finite element model of the tunnel, it is therefore necessary to specify the acoustic properties of the internal air medium, the tunnel walls, the pavement surface, and the inlet and outlet boundaries. These acoustic characteristics can be defined in terms of acoustic impedance or acoustic admittance, which are reciprocal to each other, expressed as Equation (5) [8]:

(5) $$A=\frac{1}{Z_{p}}$$

In the above expression, A represents the acoustic admittance, and $Z_{p}$ denotes the acoustic impedance. As described previously, the acoustic impedance at the tunnel portal was set as an absorbing boundary equal to the characteristic impedance of air, i.e., 416.5 $kg/(m^{2}\cdot s)$. This indicates that the boundary impedance is perfectly matched to that of the incident medium (air), thereby ensuring that no reflection occurs when sound waves reach the tunnel outlet. The tunnel walls are made of concrete, with an acoustic impedance of $Z_{p}$ = 798,000 $kg/(m^{2}\cdot s)$. The pavement surface is asphalt, with an acoustic impedance of $Z_{p}$ = 8,364,000 kg/$(m^{2}\cdot s).$

For the tunnel lining materials, the interior walls of most long tunnels and underground interchanges in China are composed of hard materials such as concrete, ceramic tiles, or stone, which typically exhibit low sound absorption coefficients, generally ranging from 0.1 to 0.15. The sound absorption coefficient is defined as the ratio of absorbed sound energy to the incident sound energy. It ranges from 0 to 1, where 0 indicates total reflection (no absorption) and 1 indicates total absorption. In general, materials with higher absorption coefficients are more effective at reducing reflected sound and controlling reverberant noise. The installation of sound-absorbing materials on the tunnel walls is a common noise mitigation strategy. These materials are broadly classified into porous absorbers and resonant absorbers. Porous absorbers attenuate sound energy through their interconnected pore structures [9], while resonant absorbers reduce sound wave reflections by leveraging the resonance characteristics of the material [7]. Both categories can be further subdivided into specific types to target different frequency ranges for noise control. A summary of commonly used tunnel inner wall sound-absorbing materials and their absorption coefficients across various frequencies is provided in Table 1 [19].

The pavement surface has a significant impact on noise levels in long tunnels and underground interchanges [3]. First, pavement conditions directly influence vehicle speed and stability, thereby altering the tire–road interaction and generating varying levels of tire and structural noise. Second, the acoustic properties of the pavement material, particularly its sound absorption coefficient, determine the extent of sound reflection, absorption, and attenuation within the tunnel. For example, materials with a high absorption coefficient can effectively reduce multiple sound reflections, thereby lowering the overall noise level, whereas low-absorption surfaces tend to cause noise accumulation [2]. Consequently, pavement characteristics not only affect noise generation but also strongly influence its propagation and spatial distribution, making them a critical factor in noise environment analysis and optimization design.

The acoustic properties of commonly used pavement materials are summarized in Table 2. Among the listed pavement types, porous asphalt exhibits the highest absorption coefficients across the frequency range, indicating its superior ability to absorb acoustic energy and reduce reflected noise. In contrast, dense-graded asphalt shows relatively low absorption coefficients, which may limit its effectiveness in noise reduction within enclosed tunnel environments.

#### 2.1.4. Computational Parameters and Computational Response Points

Traffic noise is generated as a continuous sound source when multiple vehicles pass successively, representing a dynamic and irregular acoustic source. However, under conditions of relatively stable traffic flow, traffic noise exhibits a certain degree of stability. Consistent with previous studies, in this work, traffic noise is simplified as a steady line source, in which vehicles are modeled as point sources, vehicle spacing is neglected, and the sources are assumed to be densely distributed along the roadway.

To analyze the sound pressure distribution across tunnel cross-sections in detail, computational response points were defined within the model. Given the symmetry of the tunnel structure and the noise sources in both longitudinal and transverse directions, the resulting noise field is also symmetric. To reduce computational cost, the tunnel centerline was adopted as the axis of symmetry, and response points were placed only on one side of the tunnel. The spacing of 50 m between cross-sections was chosen to balance computational efficiency and spatial resolution of the acoustic field. The selection of computational response points follows common practices in tunnel acoustics research, where sound pressure levels are typically evaluated along the tunnel centerline and at representative driver ear heights to reflect the noise exposure experienced by vehicle occupants [7]. The heights of the measurement points were selected to represent the ear positions of drivers in different vehicle types (1.2 m for small cars, 1.5 m for medium vehicles, and 1.8 m for heavy trucks), as well as the ground level (0 m). In addition, measurement points near the tunnel shoulder were included to represent the acoustic environment experienced by maintenance workers and emergency personnel.

**Figure 3:**
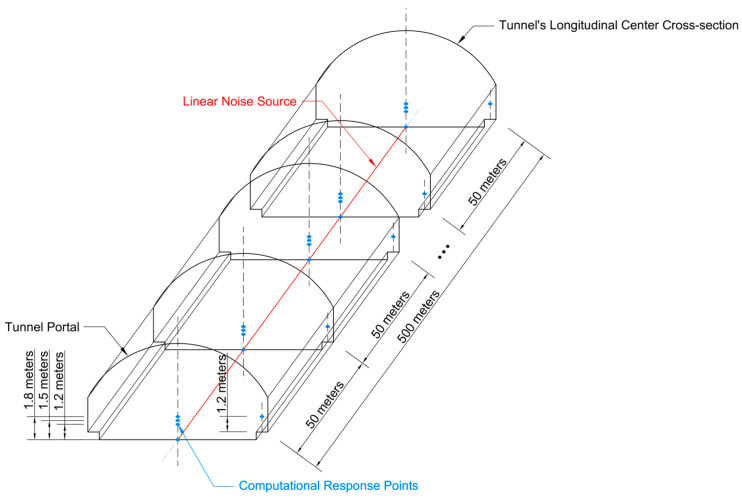
Layout of noise sources and response points.

The detailed arrangement of the computational response points is summarized in Table 3. Figure 3 illustrates the tunnel geometry, the linear noise source (located along the tunnel centerline on the road surface), as well as the positions of the computational cross-sections and response points. Through the simulation, the sound pressure levels at each response point and the overall sound pressure contours for each cross-section can be obtained.

### 2.2. Model Validation

**Figure 4:**
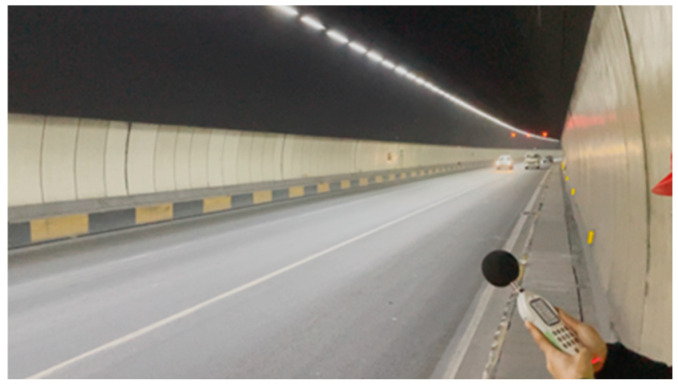
On-site measurement of tunnel noise levels.

Model validation is an essential step in acoustic simulation to ensure that numerical predictions accurately represent real acoustic environments. In tunnel noise studies, validation is typically conducted by comparing simulated sound pressure levels with field measurements in terms of both frequency spectra and equivalent A-weighted sound levels [13,20]. In this study, an on-site noise survey was conducted in the target tunnel to obtain traffic composition characteristics, equivalent noise levels, and frequency response parameters. The locations of the measurement points corresponded closely to the computational response points in the model. Starting from the tunnel entrance, measurements were taken every 50 m, with each point positioned at a height of 1.2 m above the tunnel shoulder. A Class 1 handheld multifunction sound level meter was used to continuously record noise levels at each point for 20 min, thereby acquiring the actual sound level data of the tunnel, as shown in Figure 4.

During the prescribed measurement period, the equivalent A-weighted sound level (LAeq) and the cumulative percentile levels (L10, L50, L90) were recorded at each measurement point, along with traffic flow (vehicles per hour) and the proportion of large-size, medium-size, and small-size vehicles. The accuracy of the simulation model was then evaluated by comparing the measured and simulated results in terms of both the frequency response characteristics and the A-weighted sound levels. The validation criteria were based on two metrics: (1) the difference between the measured and simulated sound pressure levels in each frequency band was required to be within 3 dB(A), which is commonly regarded as indicating good agreement in acoustic model validation studies [4,20,21]; (2) the A-weighted sound pressure level data obtained from measurements and simulations were further analyzed using the Kappa coefficient, which is commonly employed to assess the consistency between two datasets in environmental monitoring studies [6,12].

## 3. Results and Discussion

### 3.1. Model Accuracy Analysis

**Figure 5:**
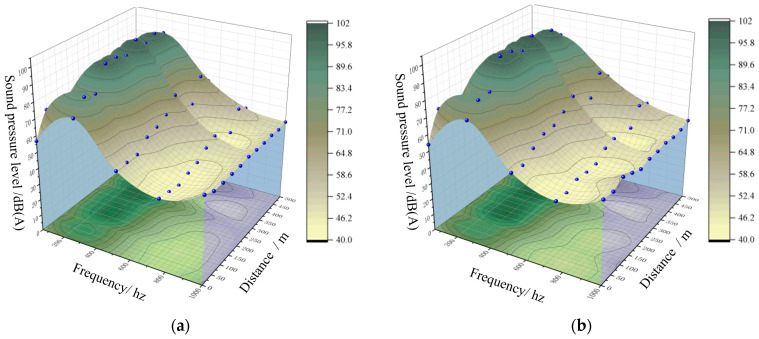
Comparison of frequency response characteristics. (**a**) Simulated results; (**b**) Field measured results. The blue dots represent the response points used in the analysis.

After constructing the tunnel model, the initial noise field was simulated. The simulated frequency response characteristics and A-weighted sound levels were compared with the field measurements to assess the accuracy of the model. The three-dimensional surfaces representing the simulated and measured frequency response characteristics are shown in Figure 5.

The differences between the simulated and measured results were further calculated, as summarized in Table 4.

**Figure 6:**
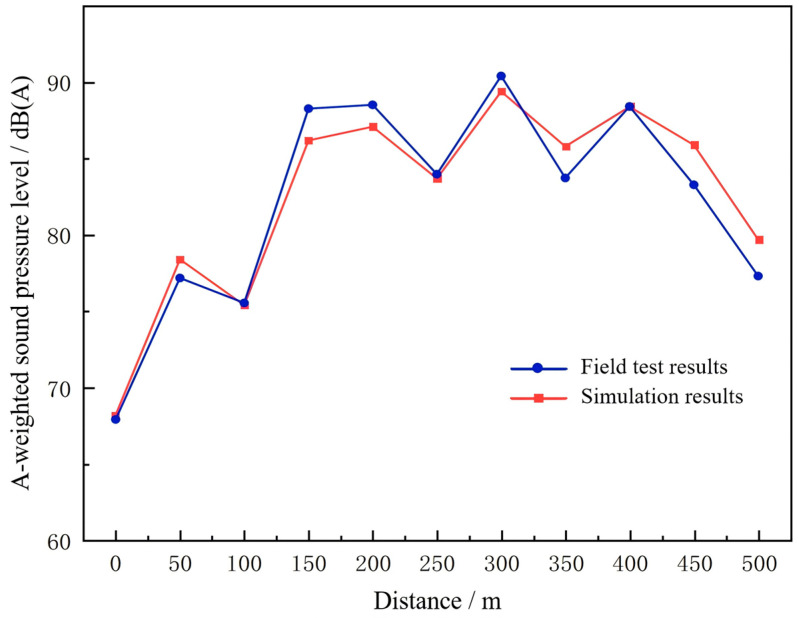
Comparison of simulated and measured A-weighted sound pressure levels.

By examining the three-dimensional surfaces shown in Figure 6, it can be observed that, across different frequencies, the variations in measured sound pressure levels along the tunnel longitudinal direction are highly consistent with the simulated results. At all positions along the tunnel length, the trends of the sound pressure levels closely follow the simulation values, with the differences between measured and simulated values remaining within 3 dB(A). This deviation is well below the allowable error range specified in the national standards for environmental noise monitoring.

The measured equivalent A-weighted sound pressure levels were further compared with the simulated A-weighted sound pressure levels, and the results are presented in Table 5.

As shown in Figure 6, the simulated A-weighted sound pressure levels exhibit trends that closely match the measured data, with the differences between the two consistently maintained within 3 dB(A). The remaining deviations between simulated and measured results may be attributed to several factors, including simplified representation of traffic noise sources, idealized boundary conditions, and uncertainties in material acoustic properties. Nevertheless, all deviations fall within the acceptable tolerance range of ±3 dB(A), confirming the reliability of the simulation model.

Furthermore, a consistency analysis using the Chi-square test was performed, yielding a Kappa value of 1. This demonstrates an exceptionally high level of consistency between the simulated and measured values, confirming the accuracy of the model and supporting its use in subsequent studies of tunnel noise characteristics.

### 3.2. Analysis of Initial Tunnel Noise Characteristics

#### 3.2.1. Overall Analysis of Tunnel Noise Characteristics

**Figure 7:**
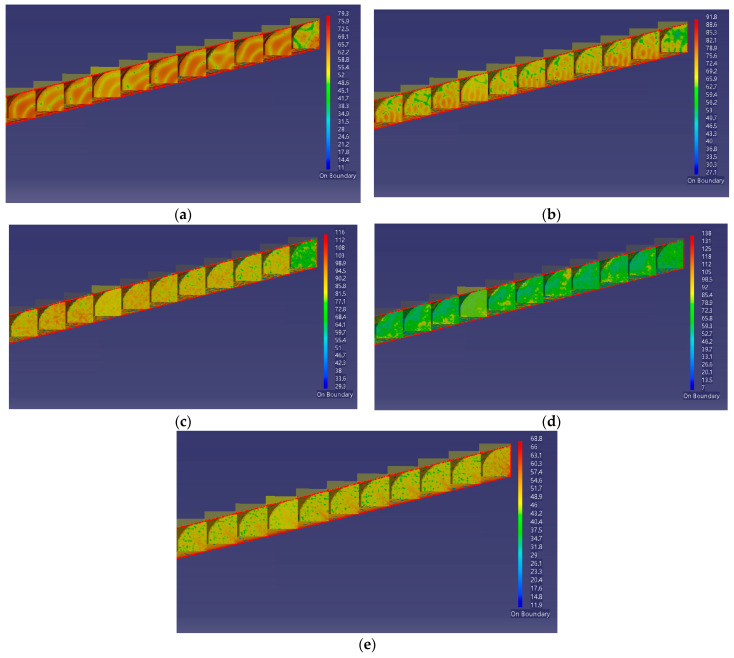
Sound pressure level contour maps at 63–1000 Hz. (**a**) Contour map at 63 Hz; (**b**) Contour map at 125 Hz; (**c**) Contour map at 250 Hz; (**d**) Contour map at 500 Hz; (**e**) Contour map at 1000 Hz.

Based on the established acoustic finite element model of the tunnel, finite element simulations were performed for five octave bands (63, 125, 250, 500, and 1000 Hz) using parallel computation. The resulting overall sound pressure distributions within the tunnel are illustrated as contour maps in Figure 7.

The sound pressure level (SPL) contour maps generated from the simulations reveal the distribution of SPLs within the tunnel at different frequencies. In the contour maps, the leftmost cross-section corresponds to the tunnel center, while the rightmost cross-section represents the tunnel exit. Due to significant variations in SPL across frequencies, the numerical legends of the contour maps are not uniform; however, the variations in SPL can be intuitively identified through differences in color intensity.

By examining the changes between cross-sections for each frequency, it is evident that SPLs at the tunnel center are substantially higher than those near the exit. Notably, the SPL distribution does not decrease monotonically from the tunnel center to the exit; in some cross-sections, SPLs are even higher than those in adjacent sections closer to the center. Within individual cross-sections, SPLs are generally higher at the road center and decrease with distance from the center. Nevertheless, some regions exhibit elevated SPLs that exceed those at the road center. Among the frequency bands, the contour maps at 125 Hz, 250 Hz, and 500 Hz display deeper colors, indicating higher noise levels. Compared to the 63 Hz and 1000 Hz bands, noise is more concentrated within these mid-frequency ranges.

These spatial and frequency characteristics indicate that tunnel noise mitigation should focus on persistent high-SPL regions and low- to mid-frequency bands, rather than assuming a uniform attenuation toward the tunnel portal.

#### 3.2.2. Noise Characteristics Along the Tunnel Centerline

**Figure 8:**
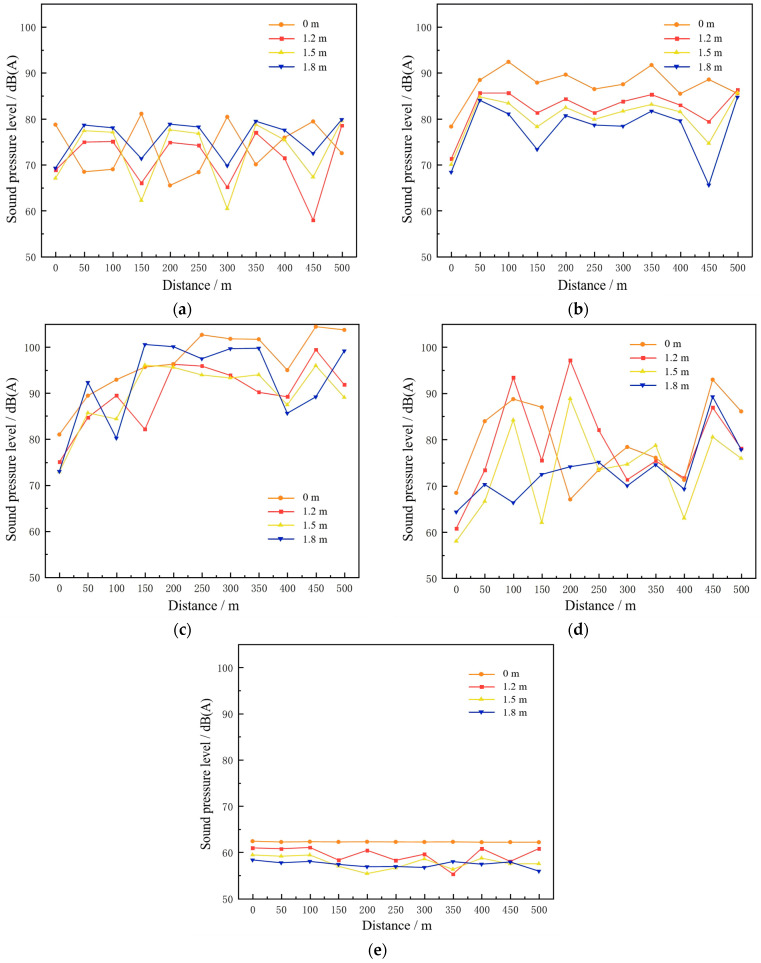
Sound pressure level along the centerline at 63–1000 Hz. (**a**) SPL at 63 Hz; (**b**) SPL at 125 Hz; (**c**) SPL at 250 Hz; (**d**) SPL at 500 Hz; (**e**) SPL at 1000 Hz.

The simulation results were organized to plot the variation curves of noise sound pressure levels along the tunnel centerline at different heights above the roadway, as a function of the longitudinal distance from the tunnel entrance. The results are illustrated in Figure 8.

The analysis reveals that at 63 Hz, the sound pressure level (SPL) exhibits a distinct periodic fluctuation, with peaks and troughs recurring approximately every 100 m. Along the tunnel centerline, the SPL increases with elevation above the road surface (1.2 m, 1.5 m, and 1.8 m). At 125 Hz, the overall SPL is higher than at 63 Hz but decreases with increasing height. At 250 Hz, the SPL exceeds that at 125 Hz and also shows a decreasing trend with elevation, though the variations among measurement points become less regular. At 500 Hz, SPL fluctuations are more irregular, with greater amplitudes between peaks and troughs, reflecting the propagation characteristics of mid-frequency noise in the tunnel. At 1000 Hz, SPL variations tend to stabilize, with the lowest overall levels, indicating a stronger attenuation effect of high-frequency noise within the tunnel.

**Figure 9:**
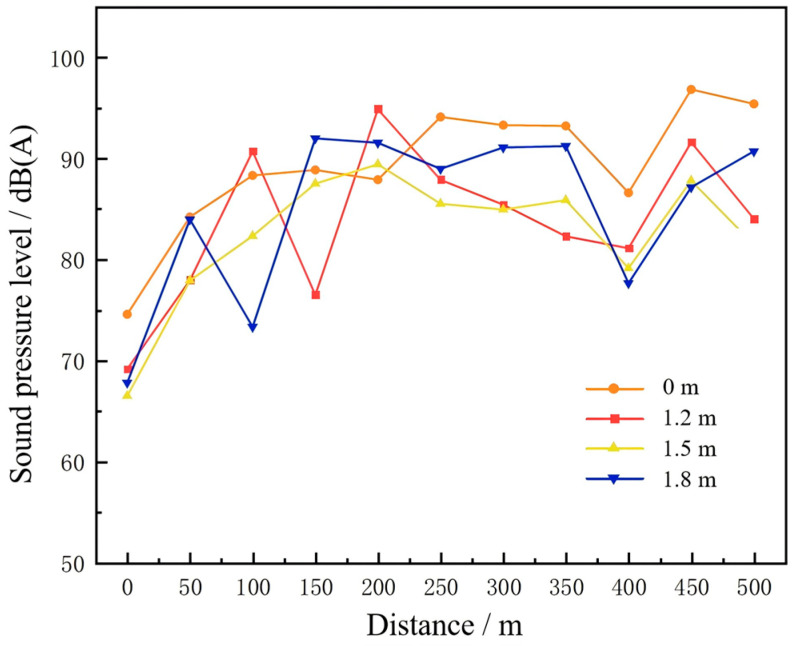
A-weighted SPL along tunnel centerline at various heights.

The calculated A-weighted sound pressure levels are shown in Figure 9. The SPL variations at different heights follow similar trends, with mean values ranked from highest to lowest as: ground level (0 m), 1.8 m, 1.2 m, and 1.5 m. This pattern reflects the vertical distribution of noise within the tunnel. When 1.2 m, 1.5 m, and 1.8 m are mapped to the auditory positions of drivers in small, medium, and large vehicles, respectively, it indicates that occupants of large vehicles are exposed to substantially higher noise levels than those in smaller vehicles. In addition, SPLs are relatively low at the tunnel portal but increase sharply within 50–100 m of entry, showing that noise intensifies progressively as vehicles move deeper into the tunnel.

This vertical variation implies that noise control measures in tunnels should consider driver position and vehicle type, as mitigation effectiveness may differ for light and heavy vehicles.

### 3.3. Noise Reduction Performance of Different Acoustic Cladding Materials

**Figure 10:**
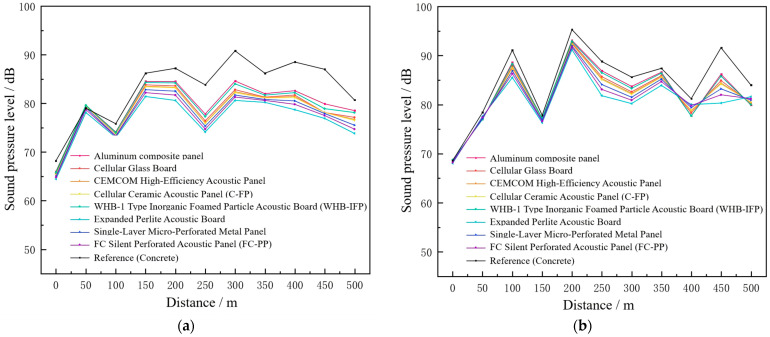
Sound pressure levels at the tunnel shoulder under different lining materials. (**a**) At the tunnel shoulder, (**b**) at the tunnel centerline.

The sound absorption coefficient of tunnel and underground interchange linings is a critical parameter influencing noise characteristics within the tunnel. Noise generation and propagation are closely related to the acoustic absorption performance of the tunnel lining, and enhancing the absorption coefficient is a key measure for reducing internal noise levels. In this study, finite element simulations were conducted to model the noise field of tunnels with different commonly used sound-absorbing materials. The resulting sound pressure levels at the tunnel shoulder and the road center under different lining configurations are presented in Figure 10.

**Figure 11:**
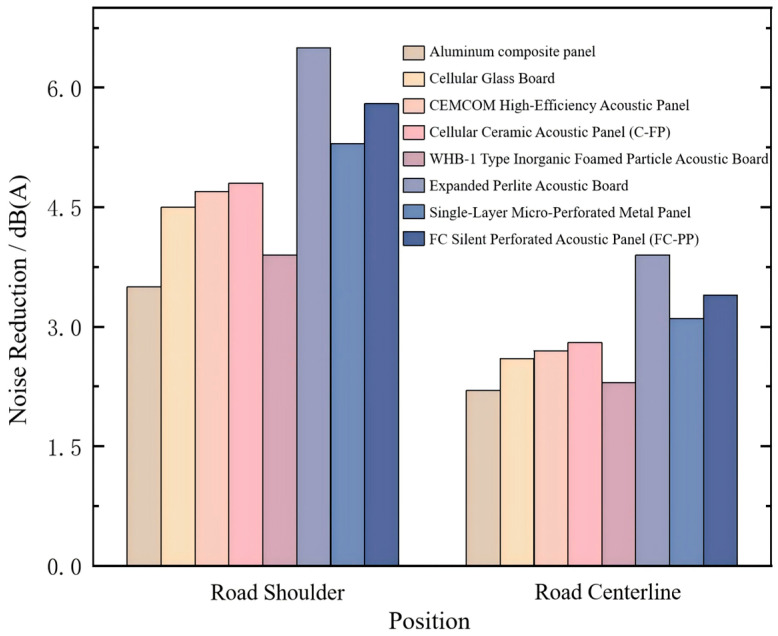
Noise reduction levels at the tunnel shoulder and centerline under different acoustic panels.

To further evaluate the effectiveness of different noise-reducing acoustic panels, the simulated sound pressure levels were compared with the baseline case without acoustic treatment (concrete lining). The corresponding noise reduction levels at the tunnel shoulder and centerline under different acoustic panel configurations are presented in Figure 11.

The results indicate that the installation of different acoustic panels provides significant noise reduction in the 150–500 m section of long tunnels and underground interchanges, whereas the effect is limited in the 0–150 m section. This is because the tunnel entrance is connected to the outside environment, leading to lower baseline sound pressure levels; thus, the absolute SPL reduction in this region is relatively small.

For noise-reducing panel selection, expanded perlite acoustic panels, single-layer micro-perforated panels, and FC silent perforated panels (FC-PP) are recommended, as they achieve substantial attenuation at both the tunnel shoulder and centerline, with average reductions exceeding 5.3 dB(A) and 3 dB(A), respectively. Among these, the expanded perlite panels perform best, yielding average reductions of 6.5 dB(A) at the shoulder and 3.9 dB(A) at the centerline. The effectiveness of these materials is primarily due to their high sound absorption coefficients in the low- to mid-frequency range. Given that the dominant noise frequencies in long tunnels and underground interchanges are concentrated between 250 Hz and 500 Hz, the absorption characteristics of these panels are well matched to the primary tunnel noise, resulting in effective noise mitigation. These findings provide a practical and efficient approach for tunnel noise control.

### 3.4. Noise Reduction Performance of Different Pavement Structures

**Figure 12:**
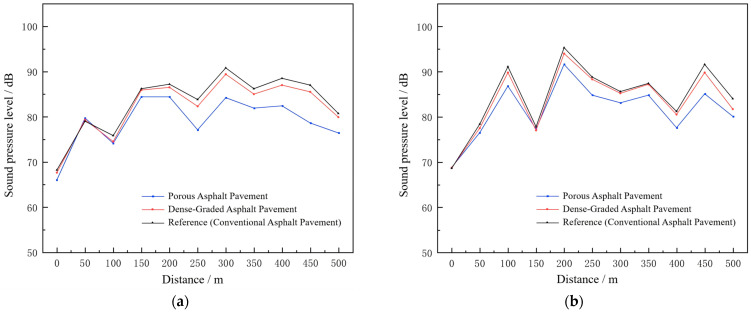
Sound pressure levels at the tunnel shoulder and centerline under different pavement types. (**a**) At the shoulder (**b**) at the centerline.

Finite element simulations were conducted using the tunnel acoustic model to evaluate the noise field under different pavement types. The resulting sound pressure levels at the tunnel shoulder and centerline for each pavement configuration are presented in Figure 12.

**Figure 13:**
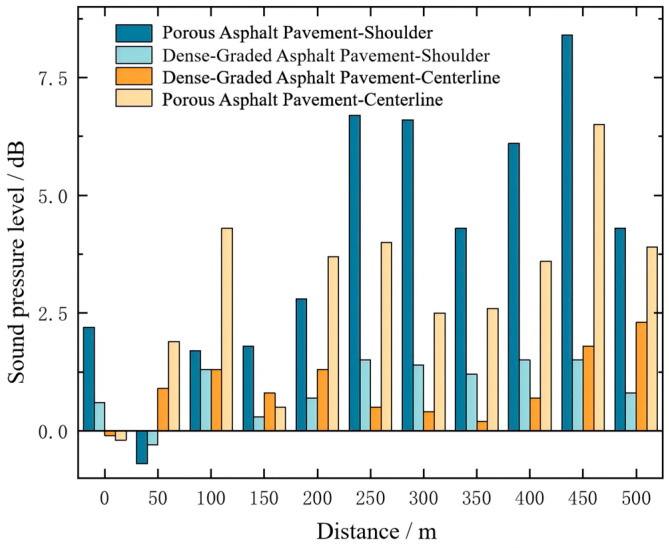
Noise reduction levels at the tunnel shoulder and centerline for porous asphalt and dense-graded asphalt pavements.

The noise reduction achieved by porous asphalt and dense-graded asphalt pavements was further compared, with the results presented in Figure 13.

A comparative analysis of the noise reduction performance of different pavement materials indicates that porous asphalt exhibits the most effective noise mitigation in tunnels. Specifically, at the tunnel shoulder and centerline, porous asphalt reduces the noise levels by an average of 4 dB(A) and 3 dB(A), respectively, which is significantly better than other pavement types. In contrast, dense-graded asphalt demonstrates weaker noise-reducing capability, with reductions of only approximately 1 dB(A) in the same regions.

The superior performance of porous asphalt can be attributed to its porous structure, which effectively absorbs and scatters sound waves, particularly in the low- to mid-frequency range where it exhibits higher sound absorption coefficients. In contrast, dense-graded asphalt shows limited noise reduction due to its low porosity and weak sound absorption. This suggests that insufficient pore connectivity restricts effective sound energy dissipation in enclosed tunnel environments.

## 4. Conclusions

In this study, an acoustic finite element model of a long highway tunnel was developed in LMS Virtual Lab and validated using in situ noise measurements. The validated model was then used to quantify the spatial and frequency characteristics of the tunnel noise field and to evaluate the noise reduction effectiveness of different lining materials and pavement types. Based on the simulation and measurement results, the main conclusions are summarized as follows.

1. By comparing the measured tunnel noise with the results obtained from the acoustic finite element model of the tunnel in LMS Virtual. Lab, the discrepancy between measured and simulated values was controlled within 3 dB(A). The high level of agreement, confirmed by the Kappa consistency test, demonstrates that the established finite element model is accurate and satisfies the required computational precision;
2. Analysis of the original tunnel noise field indicates that SPLs at the tunnel center are generally higher than at the portal, with periodic fluctuations rather than a monotonic decrease toward the exit. Both measurements and simulations reveal that the dominant noise frequencies range from 125 Hz to 500 Hz. SPLs at 1.8 m above the road surface are significantly higher than at 1.2 m and 1.5 m, suggesting that drivers of large vehicles experience higher noise exposure than those in smaller vehicles;
3. Installing sound-absorbing materials in the midsection of the tunnel provides more effective attenuation. Among commonly used lining materials, expanded perlite panels, single-layer metal micro-perforated panels, and FC quiet perforated panels (FC-PP) offer the best performance. Practical material selection should also consider cost-effectiveness for optimized tunnel noise control;
4. Comparison of different pavement types indicates that porous asphalt, owing to its high void ratio, effectively absorbs tunnel noise and achieves superior attenuation compared with dense-graded and conventional asphalt pavements. Dense-graded asphalt, with lower porosity, exhibits limited noise reduction, even less than conventional asphalt.

Overall, this study presents a noise control framework that integrates spectral analysis, targeted material selection, and multi-measure optimization, providing a practical and replicable method for tunnel acoustic design. This approach optimizes material usage and costs, enhances acoustic comfort, and reduces potential health risks and environmental impacts near tunnel entrances. Future research could consider expanding on-site measurements for different types of tunnels to enhance data representativeness and incorporate environmental factors such as weather conditions, wind speed, and humidity to aid in developing more accurate tunnel noise prediction models and effective noise reduction strategies.

## Figures and Tables

**Table 1:** Sound absorption coefficients of common noise-reduction materials. Note: “/” indicates not applicable; “—” indicates data not available.

Types of Sound Absorption Panels		Panel Thickness	Cavity Size	Center Frequency/Hz					
				125	250	500	1000	2000	4000
Panel Resonance Absorbers	Aluminum composite panel	10	/	0.04	0.07	0.16	0.60	0.80	—
Foam-Based Absorptive Materials	Cellular Glass Board	50	/	0.11	0.27	0.35	0.31	0.43	—
	CEMCOM High-Efficiency Acoustic Panel	60	/	0.21	0.33	0.83	0.83	0.94	0.87
	Cellular Ceramic Acoustic Panel (C-FP)	50	100	0.16	0.48	0.69	0.53	0.74	0.89
	WHB-1 Type Inorganic Foamed Particle Acoustic Board	50	/	0.08	0.18	0.57	0.98	0.58	0.84
Architectural Sound-Absorbing Materials	Expanded Perlite Acoustic Board	35	/	0.28	0.85	0.90	0.84	0.64	0.83
Perforated Panel Absorbers	Single-Layer Micro-Perforated Metal Panel	0.8	/	0.28	0.67	0.52	0.42	0.40	0.30
	FC Silent Perforated Acoustic Panel (FC-PP) (Perforation rate 8%)	4	100	0.53	0.77	0.90	0.73	0.70	0.65

**Table 2:** Sound absorption coefficients of common pavement materials.

Center Frequency/Hz	100	125	250	500	1000	2000	4000
Porous Asphalt Pavement	0.05	0.05	0.10	0.40	0.80	0.50	0.70
Dense-Graded Asphalt Pavement	0.02	0.03	0.03	0.03	0.03	0.02	0.02
Conventional Asphalt Pavement (Reference)	0.03	0.03	0.05	0.06	0.08	0.08	0.08

**Table 3:** The detailed arrangement of the computational response points.

Types	Design Purpose	Position	Spacing	Number
Measurement surface	SPL Contour Plot	Tunnel cross-section	50 m Distribution along the traffic stream	11 items
Measurement points	SPL	Along the tunnel centerline, at heights of 0 m, 1.2 m, 1.5 m, and 1.8 m above the ground	50 m Distribution along the traffic stream	Four series, with 11 items in each.
Measurement points	SPL	At the shoulder, 1.2 m above the road surface.	50 m Distribution along the traffic stream	11 items

**Table 4:** Deviations between simulation and field measurement of frequency response.

Distances/m	0	50	100	150	200	250	300	350	400	450	500
63 Hz	−2.8	−0.4	−2.8	−0.2	−2	1.9	1.8	1	1	0.8	1.5
125 Hz	2.7	2.6	0.7	−0.3	−2	−1.9	−2.1	1.8	−0.4	−1.6	−0.3
250 Hz	−0.1	−1.3	0.1	2.1	1.4	0.3	1.1	−2.1	0	−2.9	−2.7
500 Hz	−1.7	−2.5	2.9	1.7	0.5	1.3	−1.5	−3	2.6	0.7	1.5
1000 Hz	−3	−0.7	2.9	0.1	−2.5	−1.9	−1.6	−0.7	−2	−0.8	0.1

**Table 5:** Deviations between simulated and measured equivalent A-weighted sound levels.

Distances/m	0	50	100	150	200	250	300	350	400	450	500
Simulated results	68.2	78.4	75.4	86.2	87.1	83.7	89.4	85.8	88.4	85.9	79.7
Field measured results	67.9	77.2	75.6	88.3	88.5	84.0	90.4	83.7	88.4	83.3	77.3
Deviations	−0.3	−1.2	0.2	2.1	1.4	0.3	1.1	−2.1	0.0	−2.6	−2.4

## Data Availability

The original contributions presented in this study are included in the article. Further inquiries can be directed to the corresponding author.
